# Effect of Sleep and Biobehavioral Patterns on Multidimensional Cognitive Performance: Longitudinal, In-the-Wild Study

**DOI:** 10.2196/23936

**Published:** 2021-02-18

**Authors:** Manasa Kalanadhabhatta, Tauhidur Rahman, Deepak Ganesan

**Affiliations:** 1 College of Information and Computer Sciences University of Massachusetts Amherst Amherst, MA United States

**Keywords:** fitness trackers, cognitive performance, alertness, cognitive throughput, sleep, activity, circadian rhythms

## Abstract

**Background:**

With nearly 20% of the US adult population using fitness trackers, there is an increasing focus on how physiological data from these devices can provide actionable insights about workplace performance. However, in-the-wild studies that understand how these metrics correlate with cognitive performance measures across a diverse population are lacking, and claims made by device manufacturers are vague. While there has been extensive research leading to a variety of theories on how physiological measures affect cognitive performance, virtually all such studies have been conducted in highly controlled settings and their validity in the real world is poorly understood.

**Objective:**

We seek to bridge this gap by evaluating prevailing theories on the effects of a variety of sleep, activity, and heart rate parameters on cognitive performance against data collected in real-world settings.

**Methods:**

We used a Fitbit Charge 3 and a smartphone app to collect different physiological and neurobehavioral task data, respectively, as part of our 6-week-long in-the-wild study. We collected data from 24 participants across multiple population groups (shift workers, regular workers, and graduate students) on different performance measures (vigilant attention and cognitive throughput). Simultaneously, we used a fitness tracker to unobtrusively obtain physiological measures that could influence these performance measures, including over 900 nights of sleep and over 1 million minutes of heart rate and physical activity metrics. We performed a repeated measures correlation (*r_rm_*) analysis to investigate which sleep and physiological markers show association with each performance measure. We also report how our findings relate to existing theories and previous observations from controlled studies.

**Results:**

Daytime alertness was found to be significantly correlated with total sleep duration on the previous night (*r_rm_*=0.17, *P*<.001) as well as the duration of rapid eye movement (*r_rm_*=0.12, *P*<.001) and light sleep (*r_rm_*=0.15, *P*<.001). Cognitive throughput, by contrast, was not found to be significantly correlated with sleep duration but with sleep timing—a circadian phase shift toward a later sleep time corresponded with lower cognitive throughput on the following day (*r_rm_*=–0.13, *P*<.001). Both measures show circadian variations, but only alertness showed a decline (*r_rm_*=–0.1, *P*<.001) as a result of homeostatic pressure. Both heart rate and physical activity correlate positively with alertness as well as cognitive throughput.

**Conclusions:**

Our findings reveal that there are significant differences in terms of which sleep-related physiological metrics influence each of the 2 performance measures. This makes the case for more targeted in-the-wild studies investigating how physiological measures from self-tracking data influence, or can be used to predict, specific aspects of cognitive performance.

## Introduction

### Background

The cognitive functioning of an individual, characterized by a range of neurobehavioral metrics such as alertness, working memory, and cognitive throughput, is subject to systematic interday and intraday fluctuations [1]. These fluctuations are driven by the interplay of 3 biological processes: a circadian component *C*, a homeostatic component *H*, and a sleep inertia component *W* [2]. The circadian process *C*, governed by the circadian pacemaker (commonly referred to as the “body clock”) located in the hypothalamus, is an endogenous oscillatory process with a roughly 24-hour period. The exact phase of the circadian pacemaker in each individual is subject to variation and is determined by their chronotype—a measure of their early-bird or late-owlness [3]. This measure determines their individual time of minimum sleep propensity or peak cognitive performance during the day. Sleeping out of phase with one’s circadian rhythm—which is especially common among, but not limited to, populations such as shift workers [4]—leads to a phenomenon known as circadian misalignment. This misalignment has been shown to impact work-related fatigue [5], academic and work performance [6,7], performance on memory tests [8], etc.

In addition to this endogenous circadian rhythm, prior work has also established that an increase in the duration of wakefulness contributes to an exponential increase in sleep propensity, or homeostatic pressure [9]. This rise in homeostatic pressure *H* results in a corresponding decline in alertness and cognitive performance [10]. Other physiological measures such as heart rate variability (HRV) [11,12] and physical activity [13] have also been investigated to determine their impact on cognitive function [14]. Cognitive performance has also been found to be influenced by a variety of other factors, including sleep patterns [15], exposure to light [16], and consumption of caffeine [17].

Such fluctuations in cognitive performance are known to affect an individual’s productivity on the job and influence work-related fatigue levels [5], as well as increase the risk of occupational hazards and accidents [18]. Therefore, there is considerable interest in understanding the nature of these fluctuations in real-world settings. However, much of prior work has been done within strict experimental protocols to study the effect of individual variables while controlling for all other factors [19,20]. Thus, the effect of multiple factors on cognitive performance in real-world settings is not as well-understood.

In this work, we attempt to discern the effect of various physiological measures on cognitive performance in a real-world setting and contextualize our findings within existing theories proposed based on controlled experiments.

### Performance Prediction and Intervention Strategies

Given the widespread use of wearable devices and fitness trackers, recent efforts have explored using physiological data from these devices to characterize in-the-wild cognitive fluctuations. For example, one of the focus areas of Alphabet’s Moonshot Factory is developing a “daytime score” that goes beyond yielding data from the previous night’s sleep by harnessing this datum to tell users how prepared they are for the next day [21]. Various commercial fitness trackers are also advertised as providing metrics that can help track workplace productivity and cognitive performance [22,23].

There has been increasing work in the mobile health (mHealth) community on evaluating whether performance measures can be predicted from sleep- and circadian rhythm–based features. For instance, Abdullah et al [24] showed that alertness can be measured in the wild using a smartphone-based version of the Psychomotor Vigilance Task (PVT) [25]. Mark et al [26] studied engagement in the workplace and reported how job-related stress levels depend on the type of work being performed, and how rhythms of attention states can be identified in the workplace environment. Prior work has also looked into the effect of sleep duration and sleep debt on productivity and the use of technology among students [27]. Wahl and Amft [28] explored chronotype estimation using smartphone usage information and the 2-process circadian/homeostatic model of sleep regulation [9]. Althoff et al [29] used the speed of keystroke and click interactions on a web search engine as measures of cognitive performance and show that both metrics follow a circadian trend. Murnane et al [30] attempted to estimate sleep timings and circadian disruption from technology-mediated social interactions and, in a later study, showed that coarse-grained alertness levels can be gauged from app use features [31]. Abdullah et al [24] used sleep metrics estimated from smartphone usage to predict performance on the PVT test.

However, most studies to date have mainly focused on a single indicator of cognitive performance (eg, [24,29,31]). We argue that it is imperative to consider multiple dimensions of performance separately, because different aspects of cognitive function (eg, alertness, working memory, decision making) are known to be differently affected by factors such as sleep loss [32]. Therefore, to fully understand the effects of physiological variables on cognitive performance, one needs to take into account the multiplicity of these measures. This is especially important because different tasks may require different cognitive capabilities in order to be completed safely and responsibly. Further, many studies are constrained to a specific population, such as students or office workers, while studying cognitive performance. Our work targets a diverse population with an aim to generalize our understanding of the impact of physiological variables on 2 different cognitive measures.

### Objective of the Study

While there is substantial work on studying variations in performance, there are 3 key gaps that we try to bridge in this work: (1) connecting in-the-wild studies to theories on the influence of sleep, activity, and circadian/homeostatic rhythms on performance; (2) taking into account multiple performance measures; and (3) generalizing our analysis across diverse populations.

We examine the effects of multiple physiological parameters on different dimensions of performance in a real-world setting, and thereafter contextualize our observations in the space of existing theories. To this end, our work investigates performance across 2 axes: alertness and cognitive throughput. Through a 6-week-long research study, we collect physiological data relating to sleep and physical activity using a Fitbit fitness tracker and neurobehavioral task performance measures through a dedicated Android app. We deliberately ensure that we consider a diverse pool of participants with varying sleep and work patterns, recruiting individuals part of a regular workforce, shift workers, and graduate students. We then investigate the effects of a range of physiological and sleep-related parameters on alertness and cognitive throughput.

Our results provide insights into how sleep and activity metrics relate to work performance in different ways across alertness/cognitive throughput performance measures. We show that while alertness is sensitive to sleep duration as well as sleep stages, cognitive throughput exhibits no deteriorating effect from lack of sleep. By contrast, irregular sleep timings (ie, earlier or later bedtimes and wake-up times) indicate a significant effect on cognitive throughput, but not on alertness. We also quantify the influence of time of day, heart rate, and physical activity on both these measures.

## Methods

### Participants

We recruited 24 participants (14 female and 10 male), including 8 graduate students, 7 regular workers, and 9 shift workers, to participate in a 6-week-long research study between February and December 2019. These groups were chosen in order to ensure that our study encompasses a diverse set of participants with varying sleep and work patterns. Regular workers were individuals working jobs with a 9-to-5 schedule (or similar 8-hour daytime working hours) from Mondays to Fridays. Graduate students loosely followed a 9-to-5 weekday schedule, but reported that they also frequently worked late, occasionally worked on weekends, and had at least some flexibility in choosing their work hours. Shift workers in our study, by contrast, worked varying hours on different days of the week with shifts ranging from 8 hours to 24 hours in duration.

The study participants were recruited through convenience sampling roughly stratified by the groups mentioned above. The study was publicized through emailing lists at the authors’ institution as well as flyers posted in the surrounding area. The local police and fire departments were also contacted in an attempt to recruit emergency responders. Three of the shift workers in the study were firefighters and 1 was a police dispatcher, 3 other shift workers worked in the service industry, and 1 worked as a transit driver. This gave us the unique opportunity to study patterns of alertness and cognitive throughput among emergency responders and essential workers. All participants were between 20 and 42 years of age (mean age 28 years). One graduate student had previously been treated for insomnia and 1 shift worker had been previously diagnosed with attention-deficit/hyperactivity disorder. None of the other participants reported any history of being diagnosed with either sleep or cognitive disorders.

### Study Protocol

We used a Fitbit Charge 3 (Fitbit Inc.) and a smartphone app to collect different physiological and neurobehavioral task data, respectively, as part of our 6-week-long in-the-wild study.

#### Smartphone-Based Alertness and Cognitive Throughput Measures

Prior studies such as [32] have found that sleep deprivation can have varying effects on tasks that require sustained information processing as opposed to vigilance-based tasks. Therefore, we elected to study 2 fundamentally different aspects of human performance: alertness and cognitive throughput. We collected these metrics using an Android app to measure in-the-wild cognitive performance that was based on the toolkit produced by Dingler et al [33].

The app includes a questionnaire asking participants to rate their subjective sleepiness levels on the Karolinska Sleepiness Scale [34], and whether they had consumed a caffeinated drink in the last hour. This is followed by a task battery comprising the Psychomotor Vigilance Test (PVT; [25]) to measure alertness and an Addition Test (ADD; [35,36]) to measure cognitive throughput.

The PVT is a standard tool used to measure momentary alertness consisting of a 10-minute reaction time test that presents test takers with a visual stimulus at random intervals. The user has to press a button in response to these stimuli, and the response time is used as an objective marker of momentary alertness. Previous research has also validated the use of shorter, smartphone-based PVT tests [37-39], prompting us to use a 2-minute version of the test administered through the Android app.

Similar to prior studies such as [4,24], we studied fluctuations in alertness in terms of percentage deviation from the individual’s response time at baseline, or relative response time. Because each PVT task session consists of multiple stimuli that the participants respond to, we first compute the median response time MRT*_s,p_* for each session *s* completed by participant *p*. In doing so, we ignore all instances of false clicks where the participant taps the screen before the stimulus appears. We then discard all sessions where MRT*_s,p_* exceeds 800 ms (in comparison, the standard threshold to classify a reaction as a lapse is 500 ms [40]). Further, we remove sessions where MRT*_s,p_* falls outside 3SDs of the mean as outliers, and calculate (mean_MRT)*_p_* for each participant as the average MRT*_s,p_* across all sessions completed by *p*. Then, we calculate RRT*_s,p_* for each of their sessions as

RRT*_s,p_* = {1 – ([MRT*_s,p_*]/[mean_MRT]*_p_*)} × 100

where RRT is relative response time.

It is important to note that while higher values of response time indicate lower alertness, higher values of relative response time indicate increased alertness.

Cognitive throughput is measured using a 1-minute addition/calculation performance test (ie, ADD) [35,36], where participants sum as many pairs of 2-digit numbers as possible within a fixed duration. The user’s cognitive performance is calculated as a percentage deviation from the user’s baseline, or the relative number of additions attempted. Similar to the calculation of relative response time above, we first find NAA*_s,p_*, that is, the number of additions attempted by participant *p* in session *s*. We then remove sessions where NAA*_s,p_* lies more than 3 SDs from the mean across all sessions for that participant. We further calculate (mean_NAA)*_p_* for each participant across all sessions, and compute RAA*_s,p_* as

RAA*_s,p_* = {([NAA*_s,p_*]/[mean_NAA]*_p_*) – 1} × 100

where RAA is relative number of additions attempted.

As with relative response times, a higher RAA indicates higher cognitive throughput.

**Figure 1:**
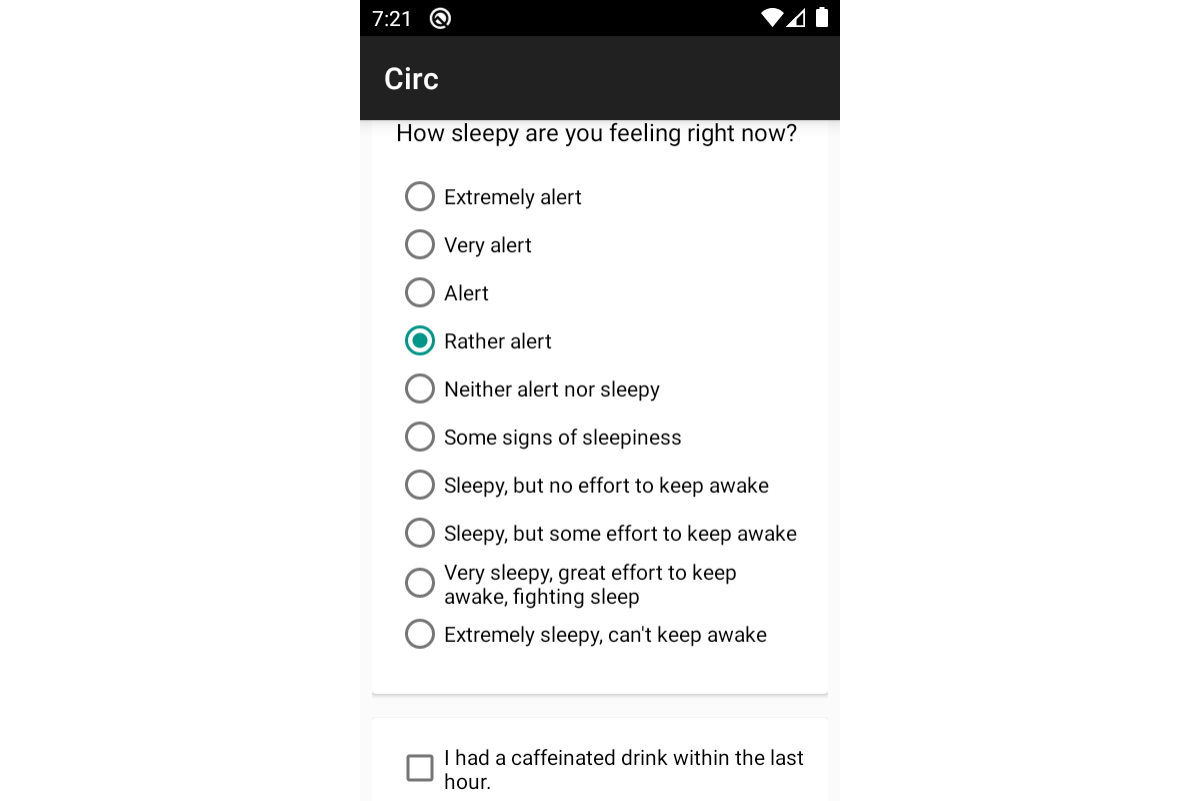
Screenshot of the Karolinska Sleepiness Scale and caffeination survey.

**Figure 2:**
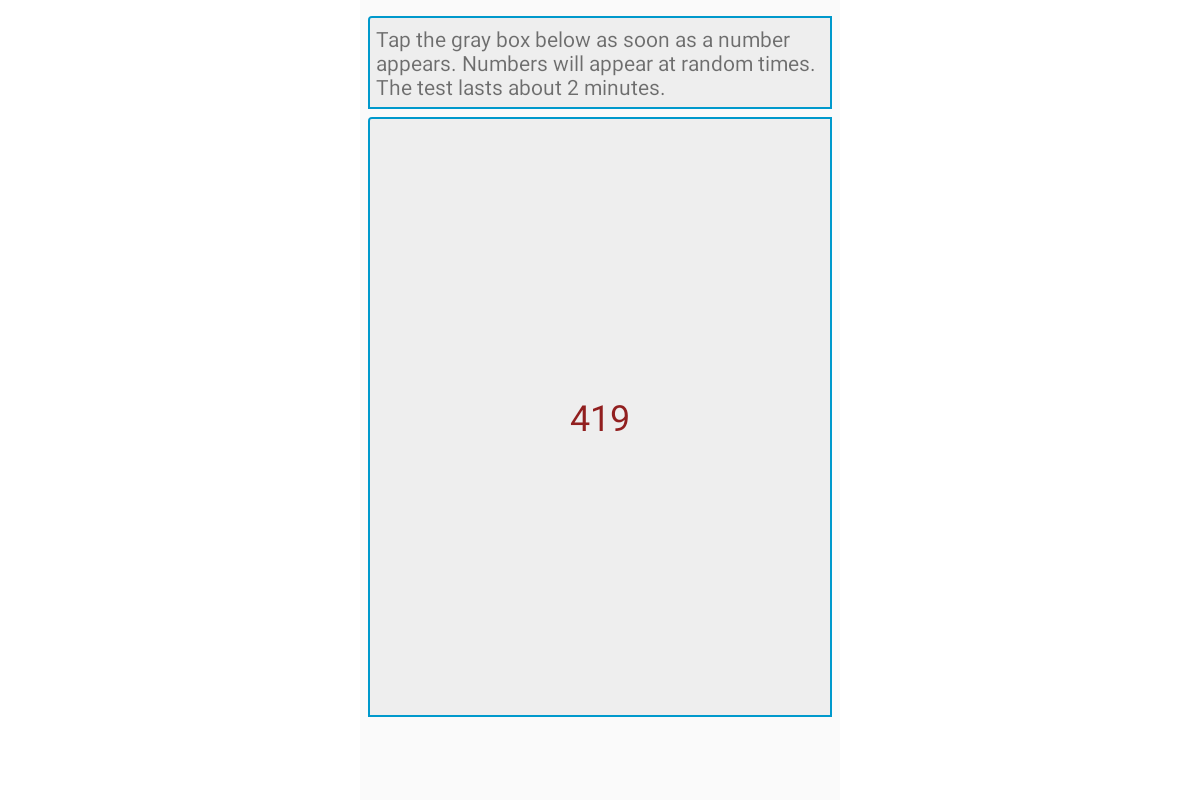
Screenshot of the PVT task.

**Figure 3:**
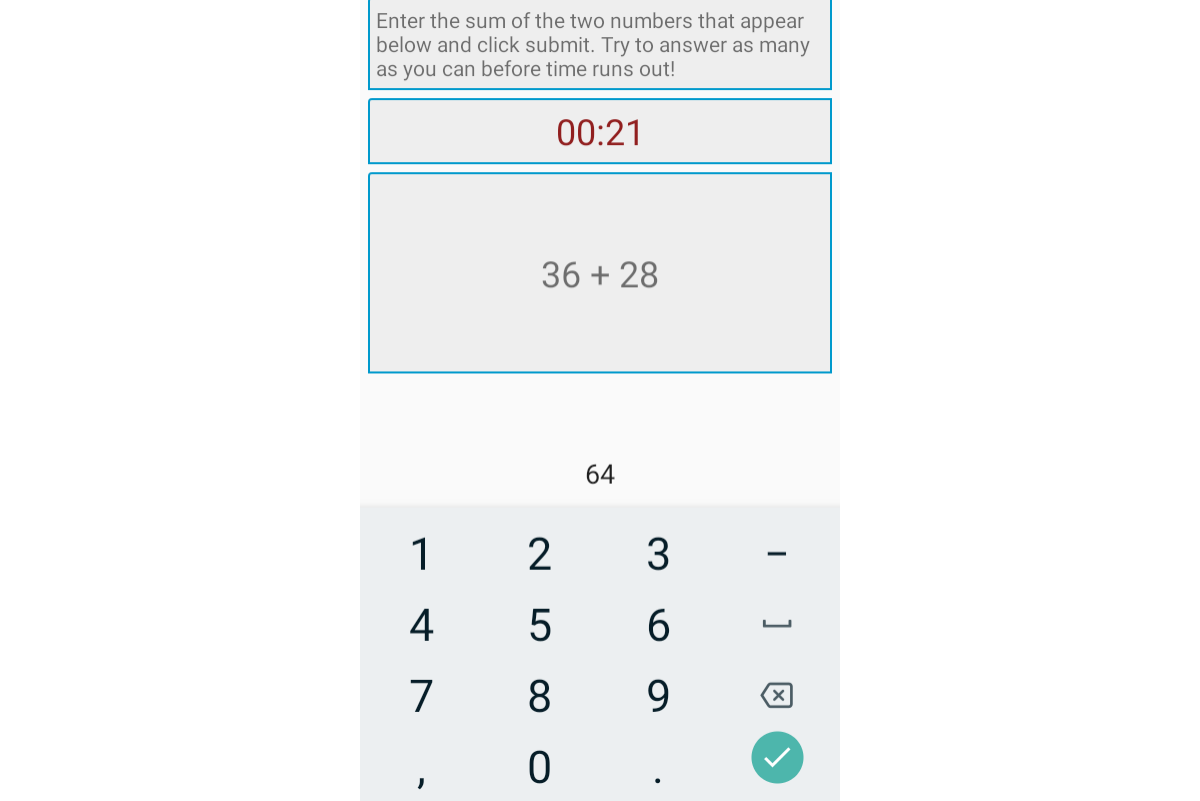
Screenshot of the ADD task.

Figure 1 shows the sleepiness and caffeination questionnaire in our Android app, while Figures 2 and 3 show the PVT and ADD tasks that participants were asked to complete. Throughout the 6-week-long study period, participants were asked to complete the task battery at least four times a day, with at least two hours between each session. As a reminder, the study app issued push notifications every 2 hours. However, participants were told to complete tasks only when they had a 5-minute distraction-free time window. We also discard the first instance of each task for each participant, as they are unfamiliar with the app at this point.

#### Physiological Measures From Fitbit Fitness Tracker

We further collected physiological data from participants using a Fitbit Charge 3, which they were asked to wear on their nondominant hand throughout the study period. Participants were specifically instructed to wear the Fitbit while they slept and while they completed the PVT and ADD tests on their phones. They were also asked to either enable auto-sync via Bluetooth on their Fitbit app or to sync their trackers periodically so as to avoid loss of data.

We obtained participants’ consent to collect data relating to their sleep, activity levels, and heart rate from Fitbit. Sleep data included the start and end time of each sleep session, time taken for sleep onset and waking up, and time spent in each stage of sleep (awake, light sleep, deep sleep, and rapid eye movement [REM] sleep) during each sleep session longer than 3 hours. Activity data included a minute-by-minute count of number of steps walked, distance covered, floors climbed, and calories burnt. Heart rate data included heart rate values each minute and the current day’s resting heart rate.

#### Compliance and Compensation

For our analysis, we only used data from participants who had completed at least forty-two PVT and ADD tasks each over the entire study period (an average of at least one task per day). We excluded 8 participants through this criterion and were left with data from 1596 PVT and ADD sessions each. We describe our data set in more detail in the “Results” section.

Participants were compensated for granting access to their Fitbit data at the rate of US $10 per week, and for completing the PVT and ADD tasks on their smartphones at US $25 per week if they completed at least four sets of tasks each day. Monetary compensation was pro-rated for the period they contributed data if it was shorter than the study duration. Participants associated with the local police and fire departments were not offered monetary compensation in accordance with the departments’ regulations. All participants were allowed to keep the Fitbit after the completion of the study. The study was approved by the Institutional Review Board of the University of Massachusetts Amherst.

### Quantifying Chronobiological Sleep Metrics

#### Sleep Data and Components Considered

We use the sleep data obtained from Fitbit to calculate 2 sets of metrics corresponding to the circadian component *C* and the homeostatic component *H*. These components, along with the sleep inertia component *W*, collectively modulate cognitive performance in humans [2]. For simplicity, we ignore *W* in our analysis.

#### Circadian Component

The phase of the circadian component is regulated by the individual’s chronotype [3]. To determine chronotype from Fitbit data, we leverage prior work on estimating individual chronotypes quantitatively using the mid-sleep point on free days (MSF), that is, the midpoint between sleep onset and wake up times on nonworking days [41]. As people tend to compensate for the sleep debt accumulated over work days by sleeping longer on free days, the quantitative measurement is typically adjusted accordingly. MSF is calculated as follows [24,41]:

MSF = MSF*_uc_* – 0.5(SD*_f_* – [N*_w_* × SD*_w_* + N*_f_* × SD*_f_*]/[N*_w_* + N*_f_*])

Here, MSF*_uc_* is the uncorrected average mid-sleep point of the participant observed across the study duration. SD*_f_* and SD*_w_* represent the sleep duration on free and work days, while N*_f_* and N*_w_* are the number of free and work days, respectively. Based on this reference marker of the individual’s circadian rhythm, an individual’s internal time (InT) is defined as the time since the individual’s MSF, or, in terms of the external (or “wall-clock”) time ExT as InT=ExT–MSF. We also quantify the misalignment between the actual mid-sleep time of an individual and their MSF, hereafter referred to as sleep shift.

#### Homeostatic Component

The sleep homeostat is responsible for building up sleep pressure during wakefulness in a sigmoidal manner and then releasing this sleep pressure during recovery sleep sessions [2,9]. One metric that captures this sleep pressure is the time since waking up from the previous sleep session.

Further, to quantify whether enough recovery sleep has been obtained to release sleep pressure, we use 2 metrics, sleep need and sleep debt. We calculate sleep debt based on individual sleep need [4]. Sleep need SN for an individual is defined as

SN=Σ(SD*_w_* × N*_w_* + SD*_f_* × N*_f_*)/Σ(N*_w_* + N*_f_*)

where SD*_w_* and SD*_f_* are the sleep durations on workdays and free days, respectively; N*_w_* is the number of work days; and N*_f_* is the number of free days. Sleep debt accrued on a given night is defined as 1−(sleep duration/*SN*).

## Results

### Data Description

The data set collected as part of our study consisted of 923 nights of sleep data, 1,032,518 minutes of heart rate data over 813 days, and 1,169,510 minutes of activity data (calories burnt, steps walked, distance traveled, and floors climbed) over 903 days. In addition to these physiological data from the Fitbit, the 24 participants in our study also completed 2059 PVT and ADD tasks through our smartphone app over the study period. In this section, we describe the collected data in further detail.

#### Smartphone-Based Neurobehavioral Task Data

**Figure 4:**
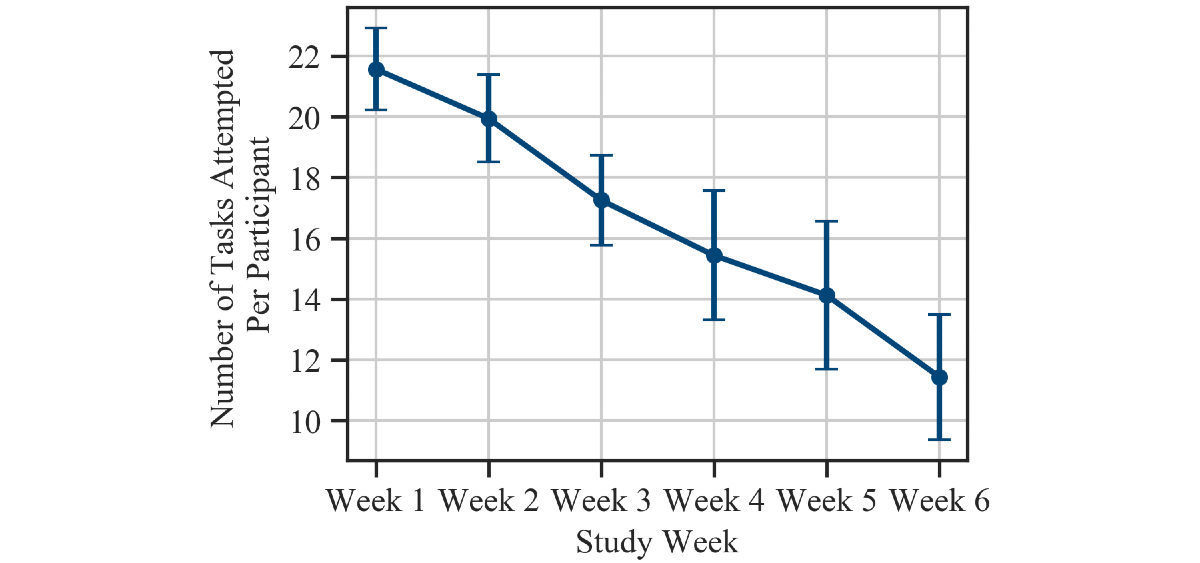
Total number of tasks attempted per participant each week of the study. The markers show the mean across all participants and the error bars indicate the standard error of the mean.

As described earlier, participants were asked to complete at least four PVT and ADD tasks each day, with at least two hours between each pair of consecutive tasks. The study app reminded users to complete these neurobehavioral tasks through push notifications every 2 hours. The average daily compliance rate across all participants was found to be 51%. We first filtered our data set to exclude all participants who had completed less than 42 PVT and ADD tasks throughout the 6-week study period (<1 task per day on average). This resulted in a data set consisting of 16 users who completed a total of 1596 tasks. The average number of tasks completed per day by these participants was 2.81 (SD 0.62). For the rest of the paper, we base our analysis on the data from these participants alone. Figure 4 shows the average number of tasks completed by these participants each week of the study. While compliance rates dropped as the study progressed, the longitudinal nature of the study ensured that all participants completed a minimum of 47 tasks or more (mean 99.75 [SD 33.06] tasks).

**Figure 5:**
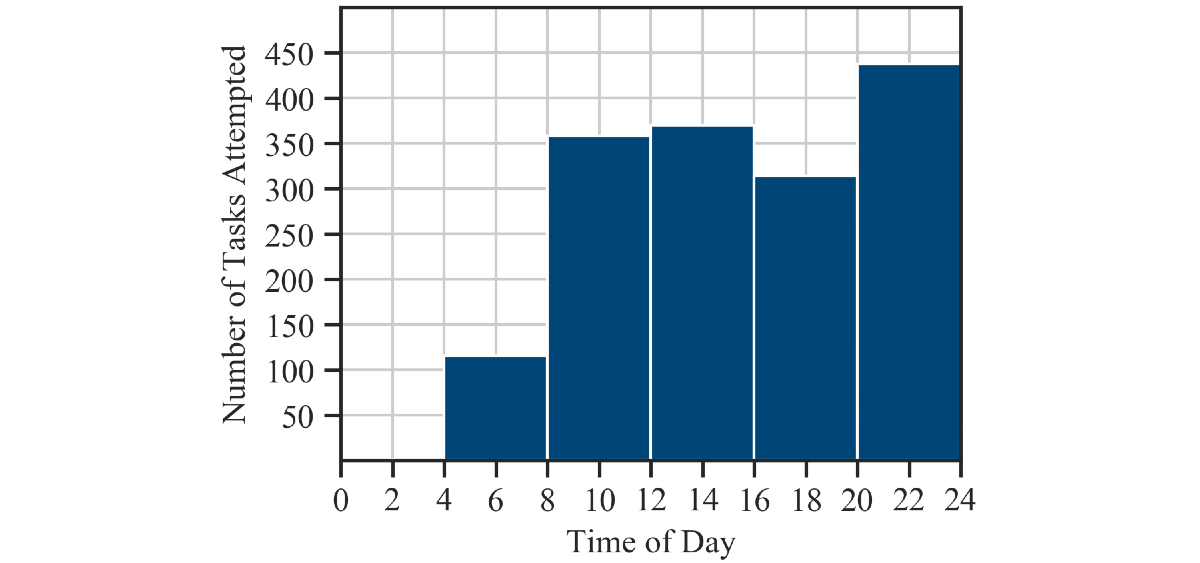
Distribution of PVT and ADD tasks attempted by time of day (binned into 2-hour intervals). Only one task was attempted in the 12AM-4AM interval.

Table 1 shows the distribution of the task completion times in terms of time of day (or wall-clock time), internal time, and time since waking up. The participants’ performance on these tasks is also reported—both PVT response times and ADD attempts were approximately normally distributed for each participant. Figure 5 shows the distribution of tasks completed by time of day.

**Table 1:** Distribution of task completion timings and performance on PVT^a^ and ADD^b^ tasks.

Task completion timings and performance		N	Minimum	Maximum	Timing, mean (SD)
**Time of task completion**					
	Time of day (hh:mm)	1596	3:22	23:58	15:34 (310.72 minutes)
	Internal time (hh:mm)	1596	0:13	23:57	11:33 (318.19 minutes)
	Time since waking up (minutes)	1596	1	1223	471.83 (309.75)
**PVT task performance**					
	Median response time (milliseconds)	1596	234.0	798.0	354.53 (90.79)
	Relative response time (%)	1596	–49.28	27.83	0.23 (8.93)
**ADD task performance**					
	Number of additions attempted	1596	7.0	30.0	17.96 (3.95)
	Relative number of additions attempted (%)	1596	–47.51	40.08	0.99 (13.46)

^a^PVT: Psychomotor Vigilance Test.

^b^ADD: Addition Test.

#### Physiological Data From Fitbit

From the 923 sleep sessions collected, we first extracted sleep sessions preceding the 1596 neurobehavioral task instances described in the previous subsection. This resulted in a subset of 556 sleep sessions across 16 participants. The features extracted from these sleep sessions include sleep timings and duration, percentages of sleep sessions spent in different sleep stages (REM, light, and deep sleep), the duration of these sleep stages, and a Fitbit-provided sleep efficiency score. We use these metrics to further calculate chronobiological measures of sleep such as sleep debt and sleep shift. We also calculate average sleep measures over the last 7 days, imputing missing nights of sleep data with the average across all previous sleep sessions. The distribution of these sleep metrics is reported in Table 2. We investigate the effect of these sleep measures on median daytime alertness and cognitive throughput on the day following the sleep session.

**Table 2:** Distribution of sleep metrics obtained from the Fitbit as well as weekly averages of the same for all sleep sessions preceding the cognitive tasks.

Distribution of sleep metrics		N	Minimum	Maximum	Mean (SD)
**Previous night’s sleep metrics**					
	Sleep duration (minutes)	556	180.0	761.0	433.12 (87.47)
	REM^a^ sleep (%)	556	2.8	34.25	18.18 (5.32)
	Light sleep (%)	556	30.04	73.71	52.92 (6.95)
	Deep sleep (%)	556	0.0	33.57	15.92 (5.1)
	Duration of REM sleep (minutes)	556	6.0	190.0	79.24 (29.04)
	Duration of light sleep (minutes)	556	79.0	429.0	228.98 (54.13)
	Duration of deep sleep (minutes)	556	0.0	157.0	68.69 (25.12)
	Number of awake periods >5 minutes	556	0.0	8.0	3.03 (1.33)
	Sleep efficiency (out of 100)	556	27	100	89.6 (14.11)
	Sleep debt	556	–0.81	0.55	0.0 (0.18)
	Sleep shift	556	–425.04	1199.21	7.1 (122.18)
**Sleep metrics over last 1 week**					
	Average sleep duration (minutes)	556	221.0	564.25	433.27 (50.53)
	Average REM sleep duration (minutes)	556	23.0	163.0	78.94 (15.59)
	Average light sleep duration (minutes)	556	84.0	310.2	228.67 (36.2)
	Average deep sleep duration (minutes)	556	28.0	123.5	69.14 (15.86)
	Average sleep debt	556	–0.17	0.19	0.0 (0.06)
	Average sleep shift	556	–311.47	202.93	6.97 (62.04)

^a^REM: rapid eye movement.

We also obtained minute-by-minute heart rate, calories burnt, steps walked, distance traveled, and floors climbed data from the participants’ Fitbit trackers, along with an estimate of the current day’s resting heart rate. Similar to sleep data, we extract heart rate and activity data corresponding to the time of completion of each of the PVT and ADD tasks. The distribution of these metrics at task time is represented in Table 3. We examined the effect of these momentary physiological measures and their aggregates over the last 60- and 10-minute intervals on both alertness and cognitive throughput.

**Table 3:** Distribution of heart rate and activity metrics obtained from the Fitbit during completion of the cognitive tasks.

Distribution of metrics			N		Minimum		Maximum		Mean (SD)
**Heart rate metrics during PVT^a^ and ADD^b^ tasks**									
	Current heart rate (bpm)	1596		45.0		131.0		79.95 (13.81)	
	Resting heart rate (bpm)	1596		47.0		88.0		66.99 (9.14)	
**Activity metrics during PVT and ADD tasks**									
	Calories burnt	1596		0.77		9.12		1.48 (0.91)	
	Distance traveled (km)	1596		0.0		0.08		0.0 (0.01)	
	Number of steps walked	1596		0		112		2.35 (10.58)	

^a^PVT: Psychomotor Vigilance Test.

^b^ADD: Addition Test.

### Effect of Sleep on Daytime Alertness and Cognitive Throughput

In order to examine the effect of sleep on interday alertness and cognitive throughput, we first calculated the daily median response times and median number of additions attempted for each individual based on each day’s completed tasks. We then calculate the relative daily response times and relative daily number of additions attempted for each day for each individual. These daily measures are calculated relative to the average scores of that individual across all days of participation (analogous to relative response times and relative numbers of additions attempted). We then examined the relationship between these relative cognitive measures and sleep features from the Fitbit. We hypothesized that interday variations in alertness and cognitive throughput can be attributed to both the sleep timings and quality of the previous night’s sleep session as well as sleep debt accumulated over a number of past sleep sessions.

To evaluate our hypothesis, we calculated the correlation between each sleep-related metric and the relative daily score. To reiterate, higher scores indicate higher alertness and cognitive throughput. In addition to sleep metrics from the previous night, we also considered cumulative sleep features over epochs of 1 week preceding the time the PVT/ADD test was administered. Because our data set consists of aggregated data from multiple participants, simple correlation can often produce spurious results due to violation of independence. To account for this as well as within- and inter-participant differences, we performed repeated measures correlation analysis [42] between our independent and dependent variables utilizing the Python package Pingouin [43]. This adjusts for interindividual variability using analysis of covariance, allowing us to draw population-level inferences while accounting for our repeated measures design.

We discuss the effect of each feature for which a significant (*P*<.05) repeated measures correlation (*r_rm_*) was observed with alertness and cognitive throughput scores. We further juxtapose our findings from a noisy, real-world data set with existing theories on the effect of sleep on cognitive processes by discussing results of prior studies, most of which have been conducted in highly controlled laboratory settings.

#### Sleep Duration

When duration of sleep obtained during a recovery sleep session on a given night falls short of the individual’s basal sleep need, it gives rise to the phenomenon of partial sleep deprivation (also known as sleep restriction or sleep loss) [44]. Sleep debt incurred in this manner can further exacerbate any existing long-term chronic sleep loss and the effects thereof [19].

Traditional sleep research has, however, focused far more on studying the effects of total sleep deprivation as compared to that of sleep restriction [44]. Total sleep deprivation occurs when individuals stay fully awake for long durations (typically 24-48 hours) with no recovery sleep obtained whatsoever. Mathematical models of cognitive performance have been heavily based on findings from such acute total sleep-deprivation studies (eg, [2,45]).

In in-the-wild studies such as ours, we are much more likely to observe chronic partial sleep deprivation, that is, a few hours of sleep loss each day, rather than acute total sleep deprivation. Chronic partial sleep deprivation is an increasingly common issue across populations due to increasing use of televisions, tablets, smartphones, laptops, or other electronic devices before bedtime [46-48]. Over prolonged exposure, the blue light from these screens suppresses the release of the sleep-inducing hormone melatonin, making it more difficult to fall asleep [49]. Therefore, it is imperative to characterize the effects of sleep debt incurred on a single, or multiple consecutive, night(s) on daytime performance measures.

Some recent studies have made efforts in this direction, studying the effects of chronic sleep loss on cognitive processes in controlled laboratory settings. For example, PVT performance has been shown to deteriorate with each consecutive day of sleep restriction [50-52]. Cognitive throughput, by contrast, has been demonstrated to show improvement across subsequent days which can be attributed to the effect of practice, implying that sleep restriction does not significantly impact cognitive throughput [50]. It has also been noted that while chronic sleep loss had a deteriorating effect on the ability to ignore distracting stimuli due to lower arousal levels, participants were able to overcome such effects on more cognitively complex logical reasoning tasks with additional effort [53].

The effect of recovery sleep to alleviate sleep debt–induced decline in performance has also been studied. PVT performance has been shown to improve with increasing hours of recovery sleep obtained, though there is some disagreement on whether this improvement is linear [54] or saturating exponential [55].

Table 4 shows the results from our study—we see that higher alertness sessions are observed following longer sleep duration over the previous night (repeated measures correlation *r_rm_*=0.17, *P*<.001) and lower accumulated sleep debt over the previous week (*r_rm_*=−0.1, *P*<.001). By contrast, we failed to observe any significant correlation between sleep duration–related metrics and cognitive throughput (*P*=.92 for sleep duration, *P*=.13 for previous week’s sleep debt). This suggests that there is validity to the theory that individuals may be able to overcome any detrimental effects of sleep debt on more cognitively challenging tasks by potentially expending more effort (see [53]). Interestingly, this behavior has also been observed in the context of total sleep deprivation, where the “controlled attention model” [32] posits that tasks that are not intrinsically engaging or challenging are more affected by sleep loss. It is also worth investigating whether there continues to be no significant detrimental effect of cumulative sleep debt over a longer period on cognitive throughput.

**Table 4:** Effect of sleep duration on daytime alertness and cognitive throughput.

Feature	Effect on alertness		Effect on cognitive throughput	
	*r*_rm_^a^ (95% CI)	*P* value	*r*_rm_ (95% CI)	*P* value
Previous night’s sleep duration	0.17 (0.08 to 0.25)	<.001	−0.004 (−0.09 to 0.08)	.92
Average nightly sleep debt incurred over previous week	−0.16 (−0.24 to −0.08)	<.001	0.06 (−0.02 to 0.15)	.13

^a^*r_rm_*: repeated measures correlation coefficient.

#### Sleep Timing

Prior work has shown that sleeping in late, that is, a later sleep end time, can lead to higher sleep onset latency on the following night as well as higher daytime fatigue and sleepiness on subsequent days [56]. However, its effect on higher-order cognitive functions has not been studied previously. Even with a fixed wake-up time, a delayed bedtime leads to a sleep phase shift, which has been shown to produce a shift in salivary dim light melatonin onset—a marker of one’s endogenous circadian rhythm [57]. We hypothesize that this circadian shift might adversely impact cognitive performance similar to a shift in wake-up times. As described earlier, sleep shift is calculated with respect to the participant’s individual chronotype, or MSF.

Table 5 shows the findings from our study, which indicate that a phase shift in sleep sessions toward a later mid-sleep time corresponds to a decline in cognitive throughput the following day (*r_rm_*=−0.13, *P*<.001). A later wake up time also has a similar effect on cognitive throughput (*r_rm_*=−0.09, *P*=.03). However, alertness scores were not found to be significantly correlated with change in sleep timings (*P*=.09 for sleep shift, *P*=.74 for sleep end time).

**Table 5:** Effect of sleep timings on daytime alertness and cognitive throughput.

Feature	Effect on alertness		Effect on cognitive throughput	
	*r*_rm_^a^ (95% CI)	*P* value	*r*_rm_ (95% CI)	*P* value
Sleep shift	−0.07 (−0.16 to 0.01)	.09	−0.13 (−0.21 to −0.05)	<.001
Sleep end time	0.02 (−0.07 to 0.1)	.74	−0.09 (−0.18 to −0.01)	.03

^a^*r_rm_*: repeated measures correlation coefficient.

#### Sleep Stages

There has been significant interest in understanding the effect of various sleep stages on daytime performance metrics, especially with the rising number of commercial wearables that claim to detect coarse-grained sleep stages. However, most experts advise caution in attributing performance to sleep stages inferred from these devices, and studies have shown that first-generation sleep trackers were generally quite poor at estimating sleep stages [58]. Nevertheless, prediction accuracies have shown improvement over time—for example, Fitbit Charge 2 showed 61% accuracy in detecting wake periods, 81% accuracy in detecting light sleep, 49% accuracy in detecting deep sleep, and 74% accuracy in detecting rapid eye movement (REM) sleep [59], whereas Garmin VivoFit 3 (Garmin International, Inc.) predicts deep, light, and REM sleep stages at roughly 69% accuracy rate, and predicts wake at 73% accuracy [60], which is a significant improvement over previous incarnations of these devices. As wearables get better at estimating sleep stages, it becomes more important to understand if we can leverage these insights to explain cognitive performance.

While the literature on the effect of sleep stages on cognitive performance is limited, studies have shown that electroencephalogram spindle density in non-REM sleep is a predictor of visual attention, verbal learning, and verbal fluency performance [61]. It has also been noted that light sleep, slow wave sleep, and REM sleep contribute to the recuperation of the dorsolateral prefrontal and inferior parietal cortices, which are areas involved in higher-order cognitive tasks [62].

Some studies have linked a reduction in total sleep duration specifically to a reduction in REM and stage 2 (light) sleep [63]. However, other studies have also claimed that selective REM sleep deprivation did not demonstrate changes in daytime sleepiness/alertness [64].

From Table 6, we see that there is a significant positive correlation between both REM (*r_rm_*=0.12, *P*<.001) and light sleep (*r_rm_*=0.15, *P*<.001) duration and daytime alertness. By contrast, sleep stages were not found to have a significant impact on cognitive throughput on the following day (*P*=.52 for REM sleep, *P*=.80 for light sleep). Duration of deep sleep was not found to have a significant impact on either alertness (*P*=.22) or cognitive throughput (*P*=.53).

**Table 6:** Effect of sleep stages on daytime alertness and cognitive throughput.

Feature	Effect on alertness			Effect on cognitive throughput		
	*r*_rm_^a^ (95% CI)	*P* value	*r*_rm_ (95% CI)		*P* value	
Duration of rapid eye movement sleep	0.12 (0.04 to 0.2)	<.001	0.03 (−0.06 to 0.11)		.52	
Duration of light sleep	0.15 (0.07 to 0.23)	<.001	0.01 (−0.07 to 0.09)		.80	
Duration of deep sleep	0.05 (–0.03 to 0.14)	.22	−0.03 (−0.11 to 0.06)		.53	

^a^*r_rm_*: repeated measures correlation coefficient.

### Factors Affecting Momentary Alertness and Cognitive Throughput

Having looked at the factors that influence day-to-day variations in daytime alertness and cognitive throughput, we now examine other factors that may impact momentary performance measures (ie, fluctuations in performance within a day). We specifically look into the effect of physical activity and heart rate, along with circadian and homeostatic effects.

#### Time of Day and Internal Time

Time of day has emerged as an important factor affecting multiple aspects of cognitive performance due to the circadian modulation of alertness and cognitive throughput [1]. Both alertness and cognitive throughput levels have been found to oscillate with a period approximately equal to 24 hours, with individuals achieving peak performance at similar times each day [65]. Further, it has been noted that an individual’s chronotype influences the phase of this circadian modulation (also referred to as process *C*), thus determining the exact time at which this peak is observed [66].

Based on this well-established model of circadian fluctuations in performance, we would expect to see a roughly sinusoidal variation in PVT and ADD performance based on the time of day and participants’ internal time. To examine whether such a relationship is indeed evident in our data, we fit cosinor models [67] with a period of 24 hours to relative response time and relative number of additions attempted based on both time of day and participants’ internal time.

As seen in Table 7, both alertness and cognitive throughput are influenced by the current time, exhibiting an acrophase (circadian maxima) in the morning hours even if they do not coincide exactly. It is also important to note that interindividual differences, including chronotype, also influence these rhythms, which prompts us to study the variations in performance measures with respect to individuals’ internal time. We see that the acrophase of these rhythms is reached about 13-14 hours after the individuals’ MSF.

**Table 7:** Effect of time of day on momentary alertness and cognitive throughput.^a^

Feature	Effect on alertness	*P* value		Effect on cognitive throughput	*P* value	
Time of day	Alertness varies sinusoidally with time of day, with an acrophase at 10:24 (09:26 to 11:21)	<.001	Cognitive throughput varies sinusoidally with time of day, with an acrophase at 09:19 (08:13 to 10:26)		<.001	
Internal time	Alertness varies sinusoidally with internal time, with an acrophase at 14:24 (13:20 to 15:31)	<.001	Cognitive throughput varies sinusoidally with internal time, with an acrophase at 13:20 (12:02 to 14:38)		<.001	

^a^The acrophase of the 24-hour circadian rhythm of cognitive performance is reported in hh:mm format with the corresponding 95% confidence intervals and *P* values.

**Figure 6:**
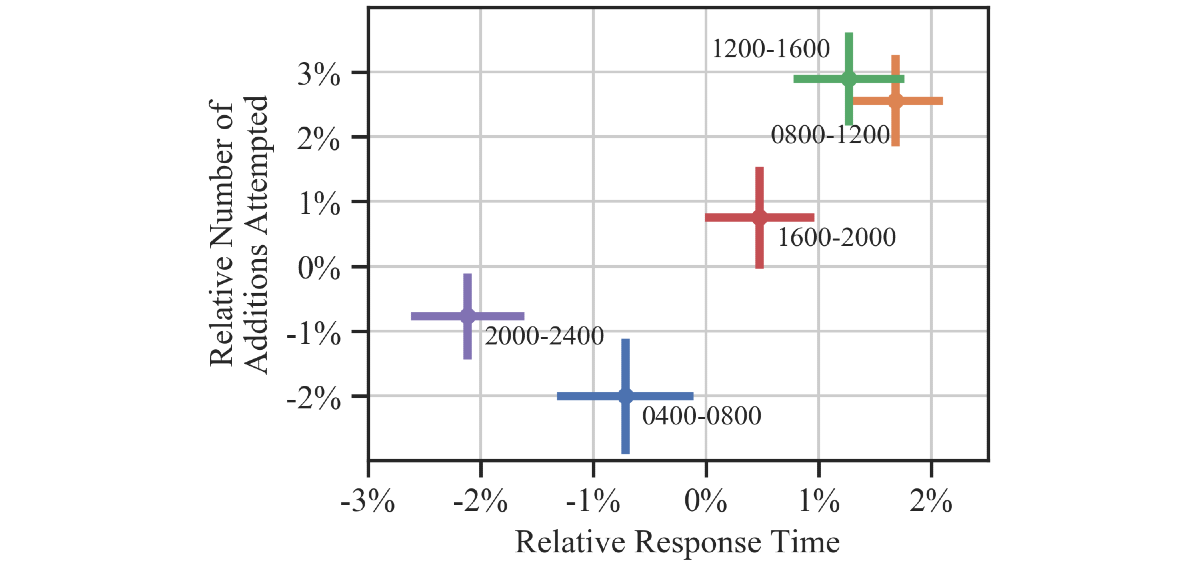
Fluctuations in cognitive performance by time of day. The x-axis represents alertness in terms of relative response times, and the y-axis represents cognitive throughput in terms of relative number of additions attempted. The dots represent the mean scores in the marked time range while the error bars represent the standard error of the mean along each axis.

Figure 6 visually illustrates the fluctuations in both performance measures across time of day. Here, we show the mean relative response times and number of additions attempted during each 4-hour time bin during the course of the day, omitting the 12:00-04:00 hours bin due to lack of data. We see that alertness increases through the day until around noon, after which it deteriorates through the evening. Cognitive throughput is lowest early in the morning, increases through the day peaking in the late afternoon, and decreases in the evening. In general, both alertness and cognitive throughput peak during the regular working hours of 08:00-16:00 hours.

#### Homeostatic Pressure (ie, Time Since Waking Up)

The homeostatic component of sleep regulation is governed by a process *H* that induces sleep pressure (or “sleepiness”) as a sigmoidal function of time since waking up from the last sleep session (typically previous night’s sleep). During sleep, this pressure is released and *H* decays in a saturating exponential manner [9]. The homeostatic component of sleep regulation is known to affect alertness, and possibly cognitive throughput, interacting with the circadian component described previously [2,15].

The effect of this sleep homeostat on daytime performance has been studied in terms of time awake in total sleep-deprivation studies, where participants are kept fully awake for very long periods (often up to 3 nights) [50]. The results of such studies generally indicate that alertness declines with an increase in hours of wakefulness [2,68,69], whereas there are conflicting theories about the effect of total sleep deprivation on more complex cognitive functions.

Traditionally, the effects of sleep deprivation were explained analogous to that of stress, based on the inverted-U model proposed by Yerkes and Dodson [70]. This “arousal” model essentially focused on the overall decline in arousal in sleep-deprived individuals in order to explain impairment of cognitive performance [71,72]. However, increasing empirical evidence showed that while performance on vigilance tasks decreased significantly across a night of sleep deprivation, performance did not vary significantly on more complex cognitive tasks [32,73]. This led to the proposal of several new theories to explain the effect of sleep deprivation on various cognitive functions.

Several researchers have proposed to single out vigilant attention as the cognitive process most susceptible to detrimental effects of sleep deprivation, while other cognitive tasks have varied sensitivity to [74], or a nonspecific effect of [75], sleep deprivation. Other studies have sought to explain their findings by claiming that tasks mediated by prefrontal cortex function are most impacted by homeostatic pressure [76,77]. Recent studies of neural activation patterns during cognitive tasks using functional magnetic resonance imaging have lent further support to this neuropsychological theory [78]. Another theory often used to explain sleep-deprivation effects is the controlled attention model [32], which posits that performance on tasks that require attentiveness and active engagement is less likely to be affected by sleep deprivation as compared to that on tasks that are not intrinsically interesting or engaging.

However, most of the aforementioned studies focus on the effects of sleep deprivation, that is, homeostatic pressure beyond at least one full day, which is much longer than typical homeostatic pressure in the working population. It is therefore still unclear how homeostatic pressure affects performance at points in time sooner after waking up, which is of greater relevance to us. To this end, we calculated the repeated measures correlation coefficient between the time since waking up from the last sleep session and performance on the corresponding PVT and ADD tasks.

As reported in Table 8, our analysis shows that alertness tends to decline with time since waking up (*r_rm_*=−0.1, *P*<.001) while no significant effect is observed on cognitive throughput (*P*=.87). This finding agrees with observations from the early stages of sleep deprivation or constant routine protocol studies (eg, [65]). This suggests that cognitive throughput may indeed be affected less by sleep homeostasis (or the effect can be overcome by effort) or may be considerably affected by sleep inertia (ie, the drowsiness felt right after waking up).

**Table 8:** Effect of homeostatic pressure on momentary alertness and cognitive throughput.

Feature	Effect on alertness		Effect on cognitive throughput	
	*r*_rm_^a^ (95% CI)	*P* value	*r*_rm_ (95% CI)	*P* value
Time since waking up	−0.1 (−0.15 to −0.05)	<.001	0.004 (−0.05 to 0.05)	.87

^a^*r_rm_*: repeated measures correlation coefficient.

#### Heart Rate

While there is limited prior research on understanding the impact of heart rate on cognitive performance, heart rhythm is generally thought to affect performance. In particular, there has been substantial work on understanding the relationship between HRV and alertness/cognitive performance [79]. HRV is considered a useful measure because it captures some aspects of the interplay between the sympathetic and parasympathetic nervous systems [80,81], which in turn has associations with the prefrontal cortex and hence cognitive performance [82].

Sleepiness is also known to relate to HRV—for example, Chua et al [83] showed that the R–R-interval power density correlates strongly with lapses on the PVT and can be used to estimate decrements in PVT performance caused by sleepiness. Henelius et al [12] report that HRV spectral power reflects vigilant attention in participants exposed to partial chronic sleep restriction. Heart rate measures have also been extensively studied in the context of fatigued driving, wherein several heart rate measures were found to be strong indicators of drowsiness under conditions of low mental workload [84]. Other work has reported that individuals with high HRV performed better on executive tasks compared to those with low HRV, but the 2 groups did not differ with regard to simple reaction time [11].

Recent work in ubiquitous computing research has also explored heart rhythm (and perceived heart rhythm) based interventions to improve cognitive performance—for example, Costa et al [85] showed that even changes in the perception of heart rate can lead to cognitive function improvement in an individual.

While our data set does not contain raw HRV information (this was not exposed by the device used for the study), we looked into the effects of coarser timescale variations in heart rate as well as direct heart rate measures on alertness and cognitive throughput. Table 9 reports the repeated measures correlation coefficients (along with the corresponding 95% confidence intervals) between various heart rate–based measures and both alertness and cognitive performance. We find that the current heart rate (measured minute-to-minute by the Fitbit) and the current day’s resting heart rate are correlated with ADD performance (*r_rm_*=0.16, *P*<.001), but a similar correlation with PVT scores was not observed (*P*=.05). Average heart rate values over epochs of 10 and 60 minutes preceding the tasks were also found to be positively correlated with task performance, with the correlations being slightly higher with ADD scores than PVT scores (*P*<.001). Higher variance in heart rate (not to be confused with HRV) over the previous hour is significantly correlated with higher cognitive throughput (*P*=.03), while higher variance in a shorter interval of 10 minutes before task time corresponds to higher alertness measures (*P*=.04). While the correlations between variance in heart rate and task performance are low, they do further underscore the importance of investigating the effects of HRV as demonstrated by prior studies.

**Table 9:** Effect of heart rate on momentary alertness and cognitive throughput.

Feature	Effect on alertness		Effect on cognitive throughput	
	*r*_rm_^a^	*P* value	*r*_rm_	*P* value
Current heart rate	0.05 (0.0 to 0.1)	.05	0.16 (0.11 to 0.21)	<.001
Resting heart rate	−0.04 (−0.09 to 0.01)	.10	0.1 (0.05 to 0.15)	<.001
Average heart rate over last 60 minutes	0.11 (0.06 to 0.16)	<.001	0.16 (0.11 to 0.2)	<.001
Average heart rate over last 10 minutes	0.09 (0.04 to 0.14)	<.001	0.16 (0.11 to 0.21)	<.001
Variance in heart rate over last 60 minutes	0.04 (0.0 to 0.09)	.08	0.05 (0.0 to 0.1)	.03
Variance in heart rate over last 10 minutes	0.05 (0.0 to 0.1)	.04	0.05 (0.0 to 0.1)	.06

^a^*r_rm_*: repeated measures correlation coefficient.

#### Physical Activity

The effect of physical activity on alertness and cognitive throughput has not been explored much in prior studies. Most in-laboratory studies prevent participants from engaging in any strenuous activity, while some sleep-deprivation studies use exercise as an additional stressor [14,86,87]. Within the latter category, studies remain inconsistent about the effects of exercise—Englund et al [14] claim that exercise did not compound effects of sleep loss, and physical activity may indeed delay any sleep loss–induced performance impairment on certain tasks. Angus et al [86] found that exercise neither increased nor decreased impairment caused by sleep deprivation. Exercise has also been found to decrease reaction times, but much less than a period of rest did [87].

The effect of physical activity on cognitive functioning of non-sleep-deprived individuals has been even less explored. Nevertheless, moderate-intensity exercise has been found to improve performance on information-processing tasks associated with sports [88,89]. These observations support the hypothesis that physically induced arousal due to exercise results in a performance improvement on cognitive tasks, which may not necessarily be explained by models of emotional arousal described previously.

Other studies suggest that physical activity can sometimes lead to higher alertness periods within break periods, but continued exertion causes this effect to wear off and may have an overall detrimental impact [13]. However, habitually engaging in moderate exercise and maintaining general fitness have also been correlated with better cognitive performance and academic achievement in children [90,91].

To investigate these effects in an in-the-wild setting, we examined whether current physical activity, or that undertaken in the recent past, has a momentary influence on either alertness or cognitive throughput. As shown in Table 10, we discovered a significant positive correlation of physical activity metrics aggregated over the previous 10- and 60-minute intervals with both PVT and ADD performance. This presents further empirical evidence in support of the arousal theory described previously.

**Table 10:** Effect of physical activity on momentary alertness and cognitive throughput.

Feature	Effect on alertness			Effect on cognitive throughput	
	*r*_rm_^a^	*P* value	*r*_rm_		*P* value
Average calories burnt over last 60 minutes	0.11 (0.06 to 0.16)	<.001	0.1 (0.05 to 0.15)		<.001
Average calories burnt over last 10 minutes	0.08 (0.03 to 0.12)	<.001	0.1 (0.05 to 0.15)		<.001
Variance in calories burnt over last 60 minutes	0.08 (0.03 to 0.13)	<.001	0.07 (0.02 to 0.12)		<.001
Variance in calories burnt over last 10 minutes	0.08 (0.03 to 0.13)	<.001	0.07 (0.02 to 0.12)		.01
Average distance traveled over last 60 minutes	0.08 (0.03 to 0.12)	<.001	0.07 (0.02 to 0.12)		<.001
Average distance traveled over last 10 minutes	0.06 (0.01 to 0.11)	.01	0.07 (0.02 to 0.12)		.01
Variance in distance traveled over last 60 minutes	0.07 (0.02 to 0.12)	.01	0.07 (0.02 to 0.12)		<.001
Variance in distance traveled over last 10 minutes	0.07 (0.03 to 0.12)	<.001	0.06 (0.01 to 0.11)		.01
Average steps walked over last 60 minutes	0.07 (0.02 to 0.12)	<.001	0.07 (0.02 to 0.12)		.01
Average steps walked over last 10 minutes	0.06 (0.01 to 0.11)	.02	0.07 (0.02 to 0.12)		<.001
Variance in steps walked over last 60 minutes	0.07 (0.02 to 0.12)	.01	0.06 (0.01 to 0.11)		.01
Variance in steps walked over last 10 minutes	0.08 (0.03 to 0.12)	<.001	0.07 (0.02 to 0.12)		.01

^a^*r_rm_*: repeated measures correlation coefficient.

## Discussion

### Principal Findings

Our results indicate that performance on less engaging tasks that require sustained attention and that on tasks which are inherently more challenging are affected differently by various components of change in sleep patterns. Alertness is significantly influenced by the duration of sleep (*P*<.001) as well as time spent in various stages of sleep (*P*<.001 for both REM and light sleep), whereas cognitive throughput is moderated by phase shifts in sleep relative to an individual’s internal circadian rhythm (*P*<.001). We also find that higher heart rate and physical activity preceding cognitively demanding tasks are positively correlated with better performance on said tasks.

### Implications

#### Performance as a Multidimensional Metric

Our findings make a strong case for treating performance as a multidimensional metric and evaluating individuals’ performance on multiple axes independently. In day-to-day societal functioning, different roles require different combinations of cognitive abilities in order to be responsibly and efficiently undertaken. For example, driving has been associated with a high need for vigilant alertness [52], whereas medical and emergency rescue personnel may rely more heavily on higher-order cognitive functions [92]. It is evident from our analysis that alertness and cognitive throughput are affected differently by different sleep-related variables, even though they covary similarly with respect to heart rate and physical activity. Thus, the ability to perform different tasks may be hindered differently due to the same changes in sleep patterns—a phenomenon that should be taken into account while predicting workplace performance, switching control of processes between human operator and automated assistants (in self-driving cars, for example), shift worker duty scheduling, etc.

#### Linking Self-Tracking Data to Actionable Insights

With commercial wearables claiming to accurately track an increasing number of physiological variables, there has been a growing interest in exploring the utility of these measures in drawing useful conclusions about users’ physical, mental, and cognitive states. Researchers are continually seeking to answer the question of what can be learned from self-tracking data, and how this knowledge can be leveraged to close the loop by providing actionable feedback to the user [93].

Consumers of commercial wearable fitness trackers are seldom aware of distinctions in performance measures such as those described in this work, and manufacturers of wearable devices make frequent claims about how users can incorporate positive behavioral changes in their lifestyles based on metrics reported by these devices. For instance, Fitbit itself reports on its blog that exercise can boost happiness and engagement, thereby increasing productivity [23]. It also outlines steps to track an afternoon drop in attentiveness through the Fitbit app using sleep and activity data, encouraging users to then administer self-timed interventions such as caffeine [94]. The company also advises tracking and improving sleep to improve workplace productivity [22], though productivity is very loosely defined. The Fitbit app also provides personalized sleep insights, such as, “You sleep a bit better on nights after a run. It’s subtle, but you spend 5 fewer minutes being restless/awake on those nights” [95]. However, the implications of these correlations are unclear—how does 5 fewer minutes of being restless/awake impact your day?

Our work shows that more comprehensive in-the-wild studies are required in order to meaningfully answer such questions. These studies should focus on understanding the effect of physiological data on specific, well-defined measures of daytime productivity and well-being, so that users can receive targeted feedback on optimizing the individual measures that are personally of most consequence to them.

### Limitations

Because our objective involved studying the effect of sleep-based metrics on cognitive performance, we opted for a longitudinal study wherein we could collect extensive data from a relatively small participant pool. A longitudinal study allowed us to capture a range of intra-individual variations and to get a better sense of our participants’ natural sleep and performance rhythms over several weeks and ensure that the chronobiological sleep metrics such as mid-sleep time on free days are more stable. However, building predictive models of cognitive performance would require further study on larger populations in order to ensure wider generalizability. As a first step, we compared the distribution of alertness and cognitive throughput across our 3 subpopulations—shift workers, regular workers, and graduate students—and found no significant differences. Thus, we are optimistic that our observations would be replicable on larger and more diverse populations.

Our results are also fundamentally limited by the accuracy of the Fitbit fitness tracker in capturing physiological metrics. Nevertheless, recent studies on these devices are promising, showing significant improvement in the validity of their inferences over time [59]. In addition, while real-world physiological data may be noisy and is likely to be confounded by several external factors, our work demonstrates that there is substantial information in these signals that can be utilized toward modeling cognitive performance.

### Conclusion

In conclusion, our work examines how metrics of sleep, activity, and heart rate that can be obtained from a commercial fitness tracker correlate with different facets of performance such as alertness and cognitive throughput. We achieve this through 2 complementary means—first, we delve into existing research in order to discern theories postulated on the basis of several controlled experiments. Second, we present insights from our own longitudinal in-the-wild study in an attempt to bridge the gap between laboratory findings and the real world.

We show that while alertness is sensitive to sleep duration as well as sleep stages, cognitive throughput exhibits no significant deteriorating effect from lack of sleep (*P*=.92). By contrast, irregular sleep timings have a significant effect on cognitive throughput (*P*<.001), but not on alertness. Both dimensions of cognitive performance show similar circadian fluctuations, but alertness is found to be more sensitive to homeostatic pressure. We also find that physical activity and heart rate have comparable effects on both alertness and cognitive throughput. The insights from our work make a strong case for treating performance as a multidimensional metric and evaluating individuals’ performance on multiple axes.

## Abbreviations

ADD: Addition Test
MSF: mid-sleep point on free days
PVT: Psychomotor Vigilance Test
REM: rapid eye movement
